# A Tribute of Respect to Josiah Trowbridge, M. D.

**Published:** 1862-10

**Authors:** 


### A TRIBUTE OF RESPECT TO JOSIAH TROWBRIDGE, M. D.

A special meeting of the Erie County Medical Society was held September 19th, at 12 M., at its rooms on South Division street. Dr. J. B. Samo, President, in the Chair, and Dr. Wm. Ring, Secretary *pro tem.*

The President said: "Gentlemen of the Erie Co. Medical Society,—It has again become my duty to convene you on the occasion of the death of another of our members—one of the most distinguished and time-honored members of this Society—Dr. Josiah Trowbridge.

If it is proper and becoming that we should meet together to commemorate those of our medical brethren, who are taken from us by death, it is *eminently* so when, as in the present instance, the subject is one who has occupied so prominent a position, both as a citizen and a physician. I do not doubt, fully impressed as we all are with the high character of the deceased, that you will make such remarks and pass such resolutions as will be appropriate to the occasion."

Dr. Moses Bristol remarked that he had been well acquainted with Dr. Trowbridge and entertained a high respect for him as a citizen and physician. The Doctor came to reside in Buffalo in 1810, while Buffalo was yet a small village, and the surrounding country but sparsely settled. He was the last of the early members and founders of this Society. He was long esteemed by the profession and the public as the head of the Faculty in this City and County. His character was well known to us all. Frank in the expression of his opinions, firm in his purposes, a warm friend, plain in his habits and tastes. He was in active practice for fifty years. In the sick room he was kind and dignified, and his unremitting care not only won the confidence of his patients as a physician, but their warmest esteem as friends. He was well versed in all the different phases of disease, and a physician of great resources in difficult and dangerous cases.

Since his retirement from practice his mind was active, though however feeble in body. His life had been one of great labor and eminent usefulness. The profession had lost one of its best members, the City one of its most honored citizens.

Dr. Charles Winne stated that probably none had known Dr. Trowbridge more intimately than himself; for him as a friend and associate he entertained the highest respect. He became acquainted with him in the year 1833, bearing to him a letter of introduction from the late Hon. A. H. Tracy. Two years after this he formed with him a partnership in business. Not long after Dr. Trowbridge retired from the firm to manage and improve his landed property. Like many others of our best and most enterprizing citizens, he was involved by the crash of 1837, and lost all he possessed. Poor, involved in debt, he resumed his practice in 1838.—Their partnership lasted until 1842. One great characteristic of Dr. Trowbridge was his honesty. To his intimate friends it is well known that he might have avoided the payment of a large debt he owed, by the technicalities of the law, and he was advised by many of his friends to do it; but he gave all his property to his assignees. By his exertions he had paid all, and though he had not died rich, he possessed *more*, an unsullied reputation, and a clear conscience. He was an active citizen, and in all public matters his opinion was eagerly sought after, and his advice highly respected. In the early part of his life he was active in politics. By his friends he was induced to become a candidate for the office of Collector of the Port; on this business he repaired to Washington; but after an interview with the authorities and learning the situation of affairs, he became disgusted with the trickery of politicians, and never afterwards could he be induced to take part in party management. In the stirring events on the frontier, during the last war he was an active participant.

He had a good New England Academic education. His medical pupilage was passed in Massachusetts, and his professional education was completed in Boston. He visited and traveled in Europe while a young man. He had a clear and vigorous intellect, and was a scrupulous observer of medical ethics. He was free of attempting to win business by disparaging the merits of his associates; of the good reputation of his brethren he was as careful as of his own. To all, his shining example was worthy of imitation.

Dr. James P. White remarked, that it was with no ordinary emotion that he arose to speak of his venerable friend and most excellent preceptor, Dr. Josiah Trowbridge. For himself he owed him a large debt of gratitude. He began the study of medicine in the office of Trowbridge & Marshall, and became a member of his family in the year 1827. What was quite unusual at that time, he received a salary for services in the office, and was also paid for teaching his sons Algebra and Latin. He was recommended by him to his friends; he gave him his confidence, and he had never knowingly violated it. He was thus enabled to complete his education. For many years after they were on intimate terms. He was the first President of the Buffalo Medical Association, and that Society done itself and him the honor of placing his portrait on its walls in the year 1846.

It was known to the public and the Society that causes which he need not mention had led to estrangement. He believed the Doctor honest in all he said or done; he could not doubt but he had been deceived and misled. None could regret more than himself these occurrences; he had made overtures for reconciliation, but they were not acceptable. Notwithstanding, these unfortunate circumstances should not prevent him from bearing testimony to the great moral worth, the scientific attainments, and his just fame as a man, a citizen, a christian and physician, nor lessen the debt of gratitude to him for his assistance in his early professional career.

Dr. White closed by offering the following resolution:

*Resolved*, That a committee of five be appointed to draft resolutions expressive of the views of the Society, and report. Also to prepare a bibliography to be presented at some future meeting of the Soceity, to be deposited in its archives, and published in the *Buffalo Medical Journal*.

The following gentlemen were appointed such Committee, (Dr. White declining to act as Chairman): Bristol, Winne, Pratt, Barnes and Loomis. The Committee offered the following resolutions, which were unanimously adopted:

WHEREAS, In the death of Doctor Josiah Trowbridge, the Erie County Medical Society has sustained the loss of its eldest and one of its most respected members, it is deemed proper that this afflicting event should be commemorated by such action as will testify to its deep sense of the loss of him, who was one of the founders of its organization, one of the most active in imparting dignity and usefulness to its proceedings, and in promoting medical science by an interchange of thought among its members.

*Resolved*, That this Society will not be uumindful of him, who among others laid its foundation, and sustained its efforts through a long period of professional activity.

*Resolved*, That duly appreciating his intelligence, his acquirements, and the high moral qualities that adorned his character, we regard his example as one worthy of emulation by all the younger members of the medical profession, who are desirous of attaining the highest excellence as men and as physicians.

*Resolved*, That in his death he has been a model of christian fortitude and resignation, bearing his sufferings with eqanaimity and inimitable patience, looking steadfastly upon the goal with trust and confidence in that God whom he worshiped in sincerity, and to whose providential care, he feared not to trust in his dying hour.

*Resolved*, That the Society proffer to the family of the deceased, its sincere sympathy and condolence in their bereavement.

*Resolved*, That the members of this Society will attend the funeral in a body, and wear the usual badge of mourning.

*Resolved*, That the proceedings of this meeting be published in the daily papers, and in the *Buffalo Medical Journal*.

The following named gentlemen were appointed bearers: Drs. Bristol, Scott, Winne, Wickoff, Mixer, Loomis, Lockwood, Gay, Rochester, Mackay.

